# m5C regulator-mediated modification patterns and tumor microenvironment infiltration characterization in colorectal cancer: One step closer to precision medicine

**DOI:** 10.3389/fimmu.2022.1049435

**Published:** 2022-12-01

**Authors:** Baoxiang Chen, Yiqing Xi, Jianhong Zhao, Yuntian Hong, Shunhua Tian, Xiang Zhai, Quanjiao Chen, Xianghai Ren, Lifang Fan, Xiaoyu Xie, Congqing Jiang

**Affiliations:** ^1^ Department of Colorectal and Anal Surgery, Zhongnan Hospital of Wuhan University, Wuhan, China; ^2^ Clinical Center of Intestinal and Colorectal Diseases of Hubei Province (Zhongnan Hospital of Wuhan University), Wuhan, China; ^3^ Hubei Key Laboratory of Intestinal and Colorectal Diseases (Zhongnan Hospital of Wuhan University), Wuhan, China; ^4^ Department of Breast and Thyroid Surgery, Zhongnan Hospital of Wuhan University, Wuhan, China; ^5^ Hubei Key Laboratory of Cell Homeostasis, College of Life Sciences, Wuhan University, Wuhan, China; ^6^ Frontier Science Center for Immunology and Metabolism, Medical Research Institute, Wuhan University, Wuhan, China; ^7^ CAS Key Laboratory of Special Pathogens and Biosafety, CAS Center for Influenza Research and Early Warning, Wuhan Institute of Virology, Chinese Academy of Sciences, Wuhan, China; ^8^ Department of Pathology, Hubei Cancer Hospital, Tongji Medical College, Huazhong University of Science and Technology, Wuhan, China

**Keywords:** 5-methylcytosine, RNA methylation, colorectal cancer, immune infiltrates, tumor microenvironment, precision medicine

## Abstract

**Background:**

The RNA modification 5-methylcytosine (m5C) is one of the most prevalent post-transcriptional modifications, with increasing evidence demonstrating its extensive involvement in the tumorigenesis and progression of various cancers. Colorectal cancer (CRC) is the third most common cancer and second leading cause of cancer-related deaths worldwide. However, the role of m5C modulators in shaping tumor microenvironment (TME) heterogeneity and regulating immune cell infiltration in CRC requires further clarification.

**Results:**

The transcriptomic sequencing data of 18 m5C regulators and clinical data of patients with CRC were obtained from The Cancer Genome Atlas (TCGA) and systematically evaluated. We found that 16 m5C regulators were differentially expressed between CRC and normal tissues. Unsupervised cluster analysis was then performed and revealed two distinct m5C modification patterns that yielded different clinical prognoses and biological functions in CRC. We demonstrated that the m5C score constructed from eight m5C-related genes showed excellent prognostic performance, with a subsequent independent analysis confirming its predictive ability in the CRC cohort. Then we developed a nomogram containing five clinical risk factors and the m5C risk score and found that the m5C score exhibited high prognostic prediction accuracy and favorable clinical applicability. Moreover, the CRC patients with low m5C score were characterized by “hot” TME exhibiting increased immune cell infiltration and higher immune checkpoint expression. These characteristics were highlighted as potential identifiers of suitable candidates for anticancer immunotherapy. Although the high m5C score represented the non-inflammatory phenotype, the CRC patients in this group exhibited high level of sensitivity to molecular-targeted therapy.

**Conclusion:**

Our comprehensive analysis indicated that the novel m5C clusters and scoring system accurately reflected the distinct prognostic signature, clinicopathological characteristics, immunological phenotypes, and stratifying therapeutic opportunities of CRC. Our findings, therefore, offer valuable insights into factors that may be targeted in the development of precision medicine-based therapeutic strategies for CRC.

## Introduction

Colorectal cancer (CRC) is a prevalent malignancy worldwide, ranks third in terms of incidence, and causes a significant burden on human health (1). Although treatment strategies have greatly improved in recent decades, CRC remains the principal cause of cancer-related mortalities, with a 5-year survival rate of 13–14% for patients with advanced CRC and distant metastasis (2). The therapies that are currently available for metastatic CRC (mCRC) include cytotoxic chemotherapy, molecular-targeted therapy, and immunotherapy (3); however, the clinical benefits of these therapeutic modalities remain unsatisfactory, mainly due to the lack of effective pre-treatment predictive biomarkers. It is therefore imperative to elucidate the molecular mechanisms underlying the tumorigenesis of CRC and identify reliable biomarkers that enable the early diagnosis and treatment response predictions for patients with CRC.

The complex crosstalk between cancer cells and the tumor microenvironment (TME) has been identified as a critical factor that drives tumor progression, metastasis, and drug resistance (4). As the “soil” of cancer cells, the TME contains various non-malignant cells, including fibroblasts, transformed cells, vascular vessels, stromal cells, and immune infiltrates (5, 6). Several studies have demonstrated that immune cells are the dominating components of the TME and that immune resistance contributes to immune evasion and tumor progression (7, 8). However, high TME heterogeneity may account for a broad range of clinical prognoses and variable responses to immunotherapies, even among the patients of the same pathological grade and clinical stage. Therefore, depicting TME heterogeneity and the associated immune infiltrates may contribute to guiding the development of precision medicine for treating CRC.

**Figure 1:**
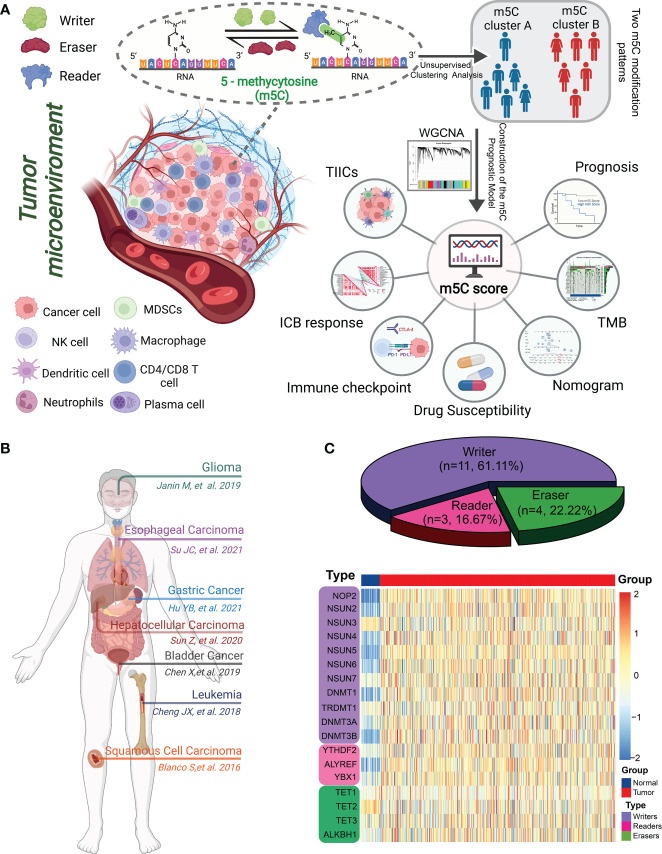
Landscape of m5C methylation regulators. **(A)** Graphical summary of the study protocol. **(B)** Overview of the m5C genes and their functions in different cancer types. **(C)** Pie charts showing the types of m5C regulators (top). The heatmap depicts the mRNA expression levels of 18 m5C regulators between normal mucosae and CRC tissues according to transcriptome data from TCGA and GTEx (bottom).

Epigenetic modifications result in heritable modulations of gene expression in the absence of a modified genomic DNA sequence. The tumorigenesis of CRC is not well-understood and has been gradually characterized based on various driver mutations and genetic and epigenetic alterations (9, 10). Several types of epigenetic regulation, including histone modification (ubiquitination, acetylation, and phosphorylation), chromatin remodeling, DNA and RNA methylation, and the expression and activity of noncoding RNA, are critical hallmarks of CRC progression (11). RNA methylation is an essential biological epigenetic process that has the functional impact on the regulation of transcriptional activation and inactivation (12). To date, more than 100 modifications have been identified for all four ribonucleotides (A, C, G, and U), including N6-methyladenosine (m6A), 5-methylcytosine (m5C), 7-methylguanosine, N1-methyladenosine, and 3-methyluracil (13, 14). Among these, m6A is the most ubiquitous and abundant post-transcriptional modification, with previous studies having identified its regulatory role in TME-specific immune infiltration (15–17). In a previous study, m6A score constructed using m6A-related genes effectively predicted the immune response and prognoses of patients with colon cancer (18). m5C is another common and well-studied RNA modification that plays a fundamental role in various biological processes, including carcinogenesis and cancer progression (**Figure 1B**) (19–24); however, the role of m5C modulators in shaping TME heterogeneity and regulating immune cell infiltration in CRC requires further investigation.

In this study, we comprehensively profiled the expression of 18 m5C regulators and identified two distinct m5C modification patterns in CRC. Additionally, we established a novel m5C scoring system using m5C-related genes identified by the weighted gene co-expression network analysis. To the best of our knowledge, these findings are the first to highlight the relationship between the m5C score and immune phenotypes, TME heterogeneity, clinicopathological characteristics, and therapeutic strategies for CRC.

## Methods

**Figure 1A** illustrates the overall workflow and mechanism diagram of this study.

### Data collection and processing

We retrieved clinical data, RNA-sequencing data, and mutation profiles for both “COAD” and “READ” from The Cancer Genome Atlas (TCGA) *via* the Genomic Data Commons portal. Copy number variation (CNV) profiles were obtained from the UCSC Xena browser, and the microsatellite instability (MSI) analysis was performed as previously described by Bonneville (25). We obtained a CRC gene expression matrix (GSE38832) with detailed clinical information from the Gene Expression Omnibus (GEO).

### Unsupervised clustering of m5C regulator genes

In total, 18 m5C genes, including 11 writers, 3 readers, and 4 erasers, were systematically analyzed using data from previous studies (26–28). Consensus unsupervised clustering analysis was conducted using the ConsensusClusterPlus package in R to explore distinct m5C modification patterns. Gene set variation analysis (GSVA) was performed using the GSVA package to calculate the enrichment score for 50 hallmark pathways from MSigDB. The differences in these pathways were analyzed between various m5C clusters using the limma package. An adjusted *P<* 0.05 and |logFC| > 0.2 were considered to be statistically significant.

### Differentially expressed genes (DEGs) and functional analysis

The limma R package was used to identify DEGs between the different m5C clusters according to the cut-off criteria of |logFC| ≥ 1 and adjusted *P*< 0.05. Gene Ontology (GO) and Kyoto Encyclopedia of Genes and Genomes (KEGG) analyses were conducted using the clusterProfiler R package based on the DEGs.

### Weighted gene co-expression network analysis (WGCNA)

The WGCNA R package was used to construct WGCNA and identify m5C cluster-related genes. First of all, the TPM data from the TCGA were tested to determine whether they were good genes or samples. Then, the filtered genes were used to construct a scale-free network by calculating the connection strength between genes. The scale independence and modules’ average connectivity were calculated using the gradient method. The appropriate power value was determined when the degree of independence was above 0.85 and average connectivity degree is relatively higher. Once the power value was determined, the scale-free gene co-expression networks were constructed. The cluster dendrogram among the modules was plotted using the ggtree package in R. The heatmap depicting the correlations between the modules and clinicopathological characteristics was generated in R using the pheatmap package. The key module with the strongest association with the m5C cluster was chosen for further analysis.

### Construction of the m5C risk score

Univariate Cox regression analysis was performed using the survival R package to identify prognostic genes. The least absolute shrinkage and selection operator (LASSO) Cox regression algorithm was implemented to minimize the risk of overfitting using the glmnet package based on the prognostic genes. Multivariate Cox regression analysis was then used to identify the candidate genes that were used to establish the prognostic m5C score. The m5C risk score was calculated using Eq. (1):


(1) m5C score = ∑(Coefi ×Expi)

where Coefi and Expi represent the risk coefficient and signature gene expression, respectively.

### Correlation analysis between clinical characteristics, the cancer stem cell (CSC) index, and MSI with the m5C score

Univariate and multivariate analysis of the clinicopathological features, including age, gender, American Joint Committee on Cancer (AJCC) stage, TNM stage, and risk score, were performed to investigate whether the m5C risk score was independent of all the other available clinical features. The relationship between the m5C score and clinical characteristics was analyzed using Chi-square test. The associations between the MSI and CSC index with the m5C score were also analyzed.

### Determining the TME immune landscape

The abundance of tumor‐infiltrating immune cells (TIICs) was quantified using the single-sample (ss) GSEA in the GSVA package. Additionally, we evaluated the differences in the gene expression of immunomodulators and immune checkpoint and effector genes between the different m5C risk groups. The anticancer immune response (cancer immunity cycle) was also evaluated between the various risk groups.

### Nomogram construction and validation

The nomogram prediction model was constructed based on the m5C risk score and clinical factors using the RMS package. Receiver operating characteristic (ROC) curves are well-known and have been used in previous bioinformatics studies; and therefore have been used to assess the discriminative performance of nomograms (29, 30).The calibration and ROC curves were used to evaluate the prediction probability and reliability of the nomogram model. The decision curve analysis (DCA) was then performed to assess the clinical performance and net benefit of the nomogram.

### Mutation and drug-susceptibility analysis

To explore the somatic mutation profiles in the different risk-score groups, the mutation annotation format data of the patients with CRC from TCGA cohort were analyzed using the Maftools package. The tumor mutational burden (TMB) score of each patient with CRC and each risk-score group was calculated and analyzed statistically. The drugs and their target information were derived from DrugBank (https://go.Drugbank.com/). The 50% inhibitory concentration (IC_50_) values of common anticancer drugs were calculated and compared in the different risk groups using the pRRophetic package.

### Tissue samples

Forty pairs of CRC specimens and adjacent normal tissues were harvested from patients at Zhongnan Hospital of Wuhan University. Written informed consent was obtained from all the participants. This study was approved by the ethics committee of Zhongnan Hospital of Wuhan University. The enrolled patients and their clinical characteristics are listed in **Supplementary Table 17**.

### RNA extraction and RT-qPCR

Total RNA was extracted from the CRC tissues using TRIzol reagent (Invitrogen, USA). cDNA was synthesized with random primers using HiScript II Q RT SuperMix (Vazyme, China). RT-qPCR was then performed using ChamQ Universal SYBR qPCR Master Mix (Vazyme). All the forward and reverse primer sequences are presented in **Supplementary Table 18**.

### Immunofluorescence

Immunofluorescence staining was performed on paraffin-embedded human CRC sections according to standard procedures. The antibodies used for immunofluorescence were anti-programmed death-ligand 1 (PD-L1; 66248-1-Ig; Proteintech), and anti-cytotoxic T lymphocyte-associated protein 4 (CTLA-4; ab19792; Abcam).

### Statistical analysis

Normally-distributed continuous variables are presented as the mean ± standard deviation and were compared using an independent Student’s *t*-test or the Mann–Whitney *U* test, whereas categorical variables were compared using Chi-square or Fisher’s exact tests. The “survcutpoint” function for the maximum rank statistic was applied to determine the optimal cutoff value of the m5C score. The survival curves for prognostic analysis of categorical variables were built using the Kaplan-Meier method, and the log-rank test was applied for statistical analysis. Spearman’s correlation coefficients and distance correlation analyses were used to assess the correlation between m5C regulators and scores with pathways related to the cancer immunity cycle or immune checkpoint blockade (ICB) response. The tumor immune dysfunction and exclusion (TIDE) analysis was performed to predict the clinical response to ICB. The survival and forestplot packages were used to perform univariate and multivariate Cox regression analyses. A time-dependent ROC analysis was performed using the timeROC package. R (version 4.1.2) was used to conduct all the statistical analyses, with *P<* 0.05 indicating statistical significance.

## Results

### Multiomics analysis of m5C regulators in CRC

**Figure 2:**
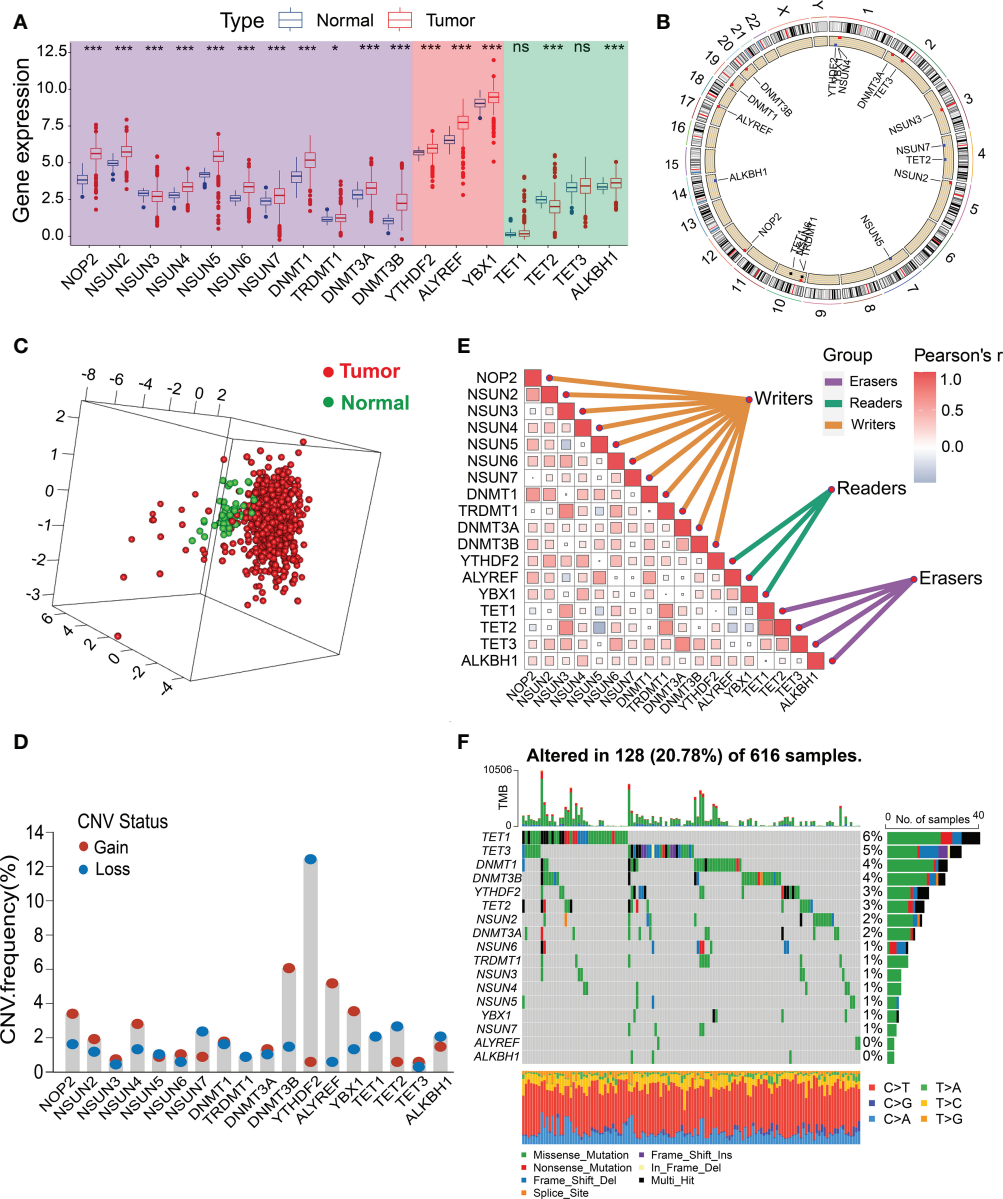
Landscape of the genetic alterations and transcriptional variations in the m5C genes in CRC. **(A)** Boxplot comparison of the differential expression levels of m5C genes between tumor and normal tissues from the TCGA-CRC dataset. **(B)** Circos plot showing the chromosomal distribution of 18 m5C genes. **(C)** PCA of the expression profiles of 18 m5C regulators. **(D)** CNV frequencies of the 18 m5C regulators. Column height represents the change in frequency. **(E)** Spearman’s correlation analysis of the 18 m5C genes from the TCGA-CRC dataset. **(F)** Mutation frequencies of the 18 m5C genes from the TCGA-CRC cohort. *P < 0.05, ***P < 0.001, ns, no significant.

We identified 18 m5C regulatory genes from the published literature, and their expression profiles in human CRC were analyzed using data from TCGA (**Figure 1C**; **Supplementary Table 1**). The expression levels of most of the m5C writers (NOP2, NSUN2, NSUN4, NSUN5, NSUN6, NSUN7, DNMT1, DNMT3A, and DNMT3B) and readers (YTHDF2, ALYREF, and YBX1) were significantly upregulated in the CRC tissues compared to those in the normal tissues, whereas the expression of the m5C eraser TET2 was downregulated in human CRC tissues (**Figure 2A**; **Supplementary Table 1**). Immunohistochemical data from the Human Protein Atlas (HPA) were consistent with the results of the transcriptomic analysis (**Supplementary Figure 1**). **Figure 2B** shows the locations of the m5C genes on their respective chromosomes. The imbalance in the expression of m5C writers, readers, and erasers may contribute to abnormal m5C modification patterns and therefore drive the oncogenesis and progression of CRC. To explore the prognostic value of the m5C regulators, we investigated the potential correlation between the gene expression levels and survival statuses of patients with CRC. The survival analysis were performed and revealed that most of the m5C genes were significantly correlated with CRC prognoses (**Supplementary Figure 2**). The principal component analysis (PCA) demonstrated that the expression of the 18 m5C regulators could be used to distinguish CRC samples from normal samples (**Figure 2C**). Moreover, the CNA analysis revealed prevalent CNV alterations in the 18 m5C genes, with most of the alterations being focused on the amplification of DNMT3B, whereas YTHDF2 showed the highest deletion frequency (**Figure 2D**). The close interaction between the m5C regulators revealed the potential value of the m5C clustering analysis (**Figure 2E**; **Supplementary Table 2**). Further investigation of the mutation patterns of the m5C genes indicated that 128 (20.78%) mutations among the CRC samples were present in the genes. TET1 showed the highest mutation frequency (6%), followed by TET3, DNMT1, DNMT3B, and YTHDF2 (**Figure 2F**). Taken together, these results demonstrate that m5C regulators may act as diagnostic biomarkers and prognostic predictors for CRC.

### Identification of m5C modification clusters and biological function analysis

**Figure 3:**
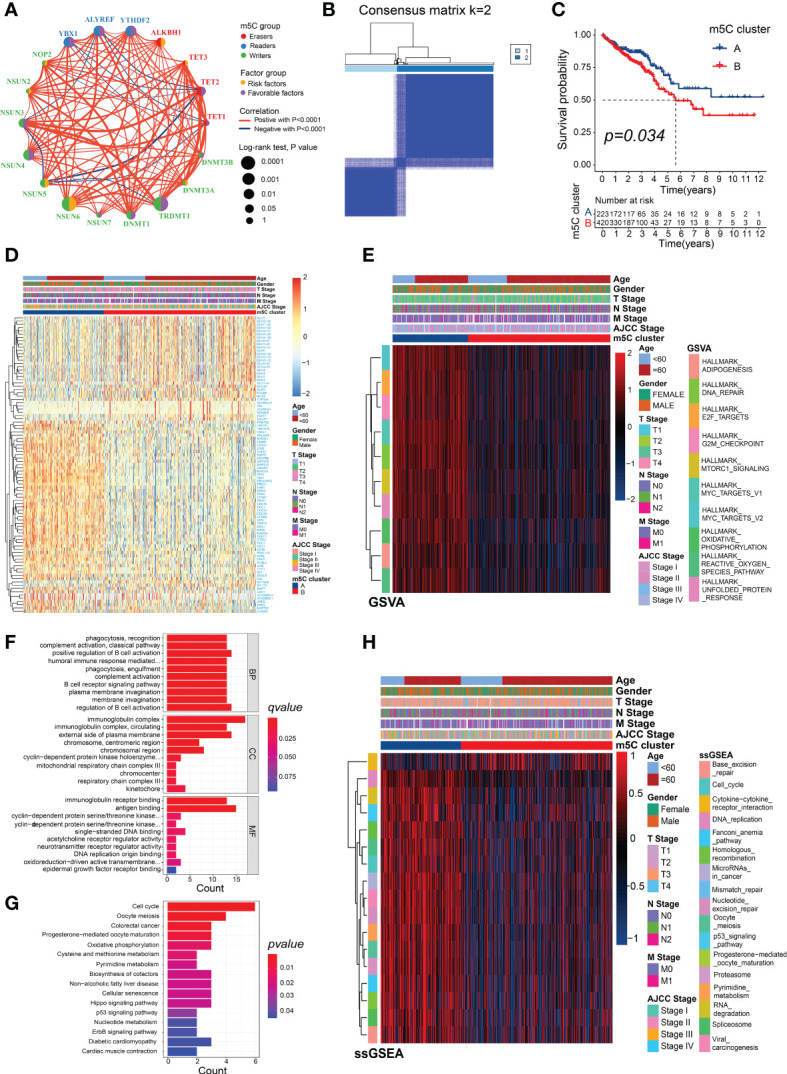
Different m5C modification patterns showing distinct biological characteristics. **(A)** Correlations and correlation coefficients between the 18 m5C regulators in CRC. Each circle represents an individual gene, and the size of the circle represents the associated prognosis. Data were generated using the log-rank test (range: 0.1–0.0001). The green or purple dots represent favorable factors or risk factors for OS, respectively, and red or blue lines indicate positive or negative correlations between the regulators, respectively. **(B)** Consensus clustering matrix (*k* = 2). **(C)** Survival analysis of the patients in the clusters generated according to m5C scores from the TCGA dataset. **(D)** Heatmap generated using DEGs between m5C clusters A and B. **(E)** Heatmap showing the GSVA analysis, which showed the activation or inhibition of biological pathways according to the m5C clusters. **(F)** GO and **(G)** KEGG analyses of the DEGs between m5C clusters A and B. **(H)** Heatmap showing the immunotherapy-predicted pathways between m5C clusters A and B.

The prognostic value, interactions, and connections among the m5C regulators in the patients with CRC are presented in **Figure 3A**. Most of these genes were risk or favorable factors and were significantly correlated with the other m5C regulators. We found significant associations between the m5C regulators from the same category as well as cross-category associations. For example, the m5C writer TRDMT1 showed a significant positive association with the writer NSUN3 and a positive correlation with the m5C eraser TET2. Unsupervised clustering analysis based on the expression of the m5C genes showed that the fewest crossovers between the CRC samples occurred at a consensus matrix *k* value of 2 (**Figure 3B**; **Supplementary Figures 3A–G**). The results of the consensus clustering were visualized using an empirical cumulative distribution function (CDF) plot and delta area plot (**Supplementary Figures 3H, I**). The Kaplan–Meier analysis of the different subtypes indicated that m5C cluster B exhibited significantly poorer prognoses than cluster A (**Figure 3C**).

The DEGs between the two m5C clusters were identified to explore their potential biological functions and were visualized in the heatmap (**Figure 3D**; **Supplementary Table 3**). There were significant differences in the enrichment scores of many of the hallmark signatures between the two clusters, including DNA repair, MYC targets, and mTORC1 signaling (**Figure 3E**, **Supplementary Table 4**). The GO analysis of the DEGs revealed significant levels of enrichment in many immune biological processes, including the regulation of B-cell activation, complement activation, positive regulation of B-cell activation, humoral immune response mediated by circulating immunoglobulin, immunoglobulin receptor binding, B-cell receptor signaling pathway, antigen binding, and immunoglobulin complex (**Figure 3F**; **Supplementary Table 5**). The KEGG analysis revealed that the DEGs were significantly enriched in several common cancer-related pathways, such as CRC, the p53 signaling pathway, Hippo signaling pathway, oxidative phosphorylation, ErbB signaling pathway, nucleotide metabolism, and pyrimidine metabolism (**Figure 3G**; **Supplementary Table 6**). More importantly, several of the pathways that were positively correlated with the ICB response, such as the DNA replication and RNA degradation pathways, and the cell cycle were enriched in m5C cluster A, whereas the cytokine–cytokine receptor-interaction pathway that was negatively associated with the ICB response was significantly enriched in m5C cluster B (**Figure 3H**; **Supplementary Tables 7, 8**). These results suggest that m5C modifications play a critical role in tumor progression and the immune regulation of the TME.

### Construction and validation of prognostic risk models based on m5C-related genes

**Figure 4:**
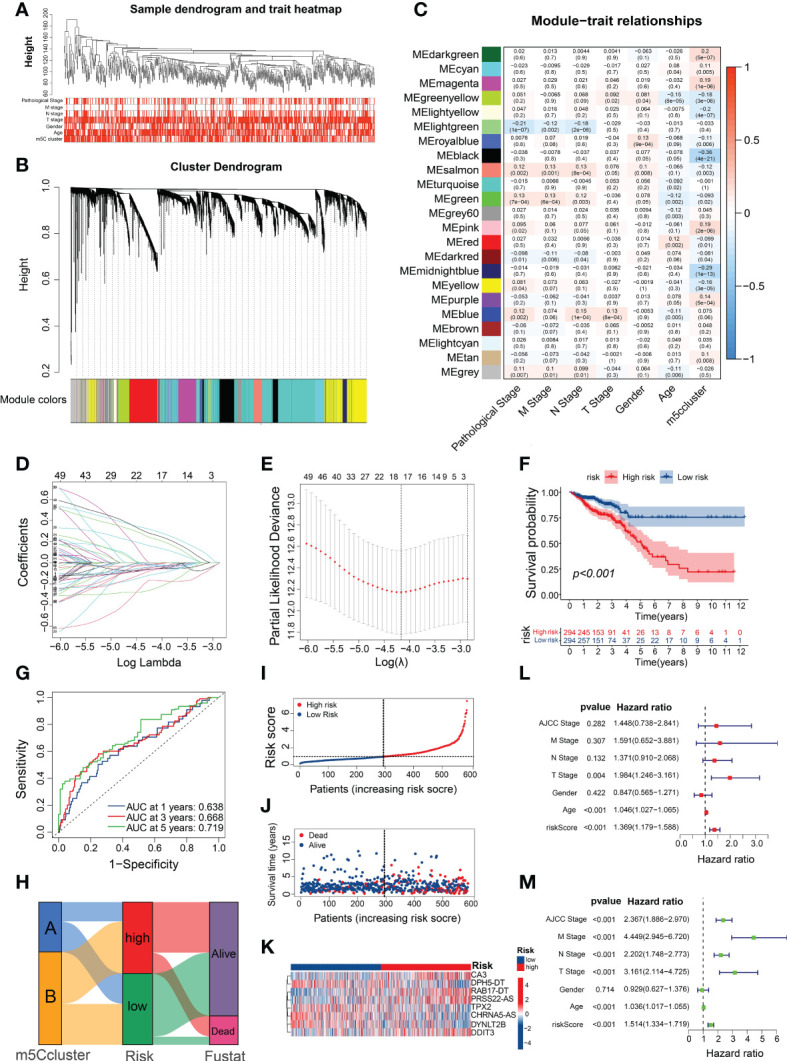
Construction of a prognostic signature using m5C-related genes. **(A)** Clustering dendrogram of the CRC samples and associated clinical traits. **(B)** Clustering dendrogram of the genes with dissimilarity on the basis of topological overlap with the corresponding module colors. **(C)** Heatmap of the association between module eigengenes and clinical phenotypes of CRC. **(D)** LASSO coefficient profiles of m5C-related genes. **(E)** Partial likelihood deviance for the LASSO coefficient profiles. **(F)** Survival analysis of the CRC patients stratified by the m5C risk score. **(G)** ROC curves for predicting the sensitivity and specificity of 1-, 3-, and 5-year OS based on the m5C score. **(H)** Alluvial diagram of subtype distributions in the groups with different m5C scores and survival outcomes. **(I, J)** Ranked dot and scatter plots showing the m5C score distribution and patient survival statuses. **(K)** Heatmap of the expression of eight m5C-related genes in the different m5C risk groups. **(L, M)** Univariate and multivariate Cox analyses of the m5C risk scores and clinical variables.

To identify m5C cluster-related modules, co-expression network was built from the expression data from TCGA-CRC. Overall, the CRC samples with intact clinicopathological information were incorporated into the co-expression analysis (**Figure 4A**). When constructing the network, we chose the power of β = 5 as the soft threshold value (**Supplementary Figure 4A**). The WGCNA identified 23 different-colored modules (**Figure 4B**), with the black module showing the highest association with the m5C cluster (**Figure 4C**). Moreover, we found that the genes in the black module were significantly co-expressed (**Supplementary Figure 4B**; **Supplementary Table 9**).

Given the essential role of m5C modification patterns in CRC tumorigenesis and the TME, we developed a prognostic signature for CRC and subsequently identified 66 prognostic m5C-related genes in the key module based on the univariate Cox regression analysis (**Supplementary Table 10**). Among them, 17 genes were screened using the LASSO Cox regression model and partial likelihood deviance (**Figures 4D, E**; **Supplementary Table 11**). We then conducted multivariable Cox regression analysis and identified eight m5C-related genes (**Supplementary Figure 4C**), which were used to build the risk model based on the Akaike Information Criterion. The prognostic risk score formula was as follows (**Supplementary Table 12**): risk score = DYNLT2B × (−0.19856) + TPX2 × (−0.33132) + DDIT3 × (0.24658) + RAB17-DT × (0.43442) + CHRNA5-AS × (−0.24444) + CA3 × (0.44152) + DPH5-DT × (−0.47732) + PRSS22-AS × (0.40269). Based on the median risk score, all the CRC patients were equally classified into high- and low-risk groups, with those in the high-risk group exhibiting evidently worse prognoses than those with low m5C score (**Figure 4F**). In addition, the predicted survival ROC curve confirmed the precise predictive capacity of the risk model, with area under the ROC curve (AUC) values of 0.638, 0.668, and 0.719 for 1-, 3-, and 5-year survival, respectively (**Figure 4G**). The risk scores of the patients in m5C cluster A were substantially lower than those of the patients in m5C cluster B (**Supplementary Figure 4D**), with the Sankey diagram indicating a relationship between the patients with CRC according to the m5C score and clusters (**Figure 4H**). The calculation and ranking of the risk score for each patient in ascending order resulted in a risk distribution plot that revealed significant decreases in survival time and increases in the mortality rate along with increasing risk scores (**Figures 4I, J**). As shown in the heatmap in **Figure 4K**, the expression levels of the eight m5C-related genes differed considerably between the various risk groups. Interestingly, most of the m5C regulators were significantly differentially expressed between the high- and low-risk groups (**Supplementary Figure 4E**; **Supplementary Table 13**). To assess whether this prognostic signature may be an independent predictor of CRC, univariate and multivariate Cox regression analyses were performed with the clinicopathological features (age, gender, AJCC stage, and TNM stage) and risk score. Compared with the other clinical features, the risk score was identified as an independent prognostic factor regardless of the univariate or multivariate analyses (**Figures 4L, M**). These results indicate that the m5C risk model may serve as a powerful prognostic indicator for patients with CRC.

### Relationship between the m5C score and clinicopathological and immunological features

**Figure 5:**
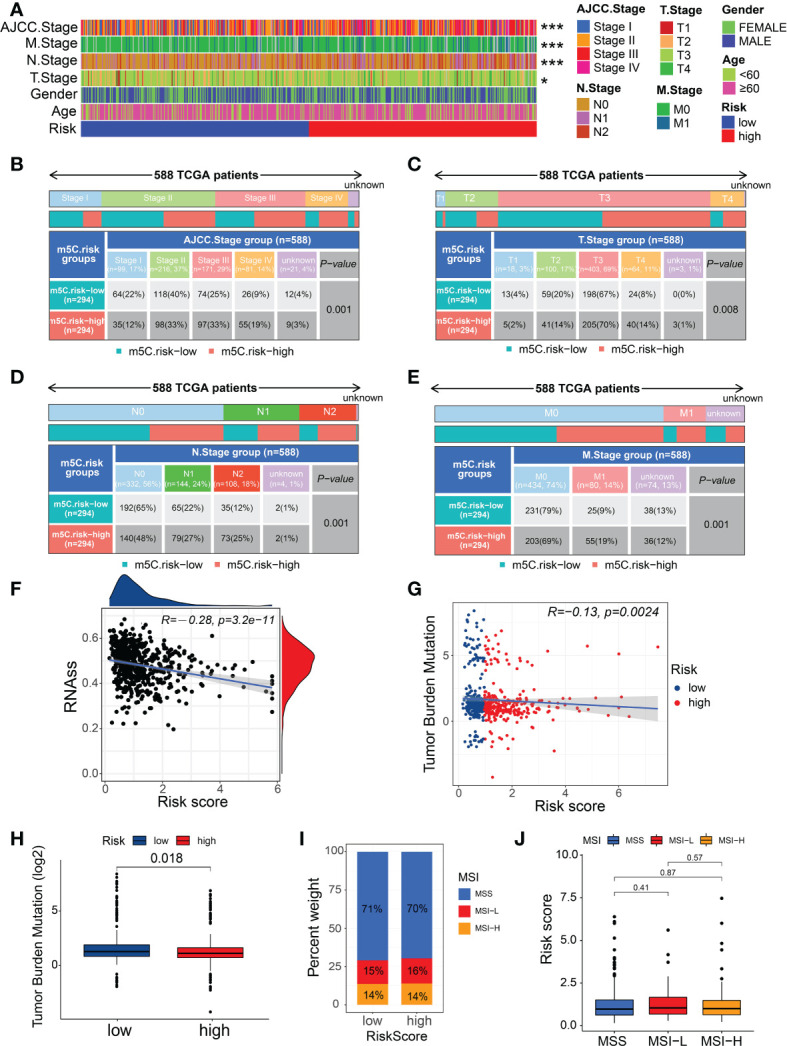
Relationship between the m5C score, clinical features, and immunological characteristics. **(A)** Heatmap of the distribution of clinical characteristics and corresponding m5C risk score in each CRC sample. **(B–E)** Heatmap and table indicating the distribution of the clinicopathological features between the high- and low-m5C-score groups. **(F)** Relationships between the m5C risk score and CSC index. **(G)** Spearman’s correlation analysis of the m5C scores and TMB. **(H)** TMB in the different m5C risk groups. **(I, J)** Relationships between the m5C risk score and MSI. *P < 0.05, ***P < 0.001.

We determined the relationship between the m5C score and clinicopathological traits of patients with CRC. The clinicopathological features related to the m5C score in the two risk subgroups are presented in **Figure 5A**. The patients with CRC at AJCC stages III–IV were mostly in the high-risk subgroup, whereas those at stages I–II were mostly in the low-risk subgroup (**Figure 5B**). Similarly, the patients with CRC who were diagnosed with T3-4, N1-2, or M+ showed significantly higher enrichment levels in the high-risk group (**Figures 5C–E**). These results indicate that the high m5C-risk score may efficiently predict advanced AJCC stages, lymphatic and distant metastases, and poorer survival in patients with CRC.

As a stem cell disease, the occurrence of CRC has been found to potentially originate from CSCs generated by intestinal stem cells escaping regulation (31, 32). We found that the m5C score showed a linear inverse association with CSC index values, suggesting that CRC patients with a lower m5C score also exhibit less stem cell differentiation and distinct stem cell properties (**Figure 5F**). A recent study confirmed the TMB to be an effective biomarker for immunotherapy (33). Moreover, cancers with high TMB may present higher levels of neoantigens, making them targets of the host immune system (34). The results of the analysis from TCGA-CRC data indicated that patients in the low-risk group showed the significantly higher TMB than those in the high-risk group (**Figure 5H**), implying that patients with a low m5C risk score tend to obtain survival benefits from immunotherapy in clinical practice. Furthermore, the Spearman’s correlation analysis confirmed a negative correlation between the m5C score and TMB (**Figure 5G**); however, no significant difference was observed in the m5C score between the different MSI statuses or in the MSI types between the different risk groups (**Figures 5I, J**).

### Correlation of the m5C score with immune phenotypes

**Figure 6:**
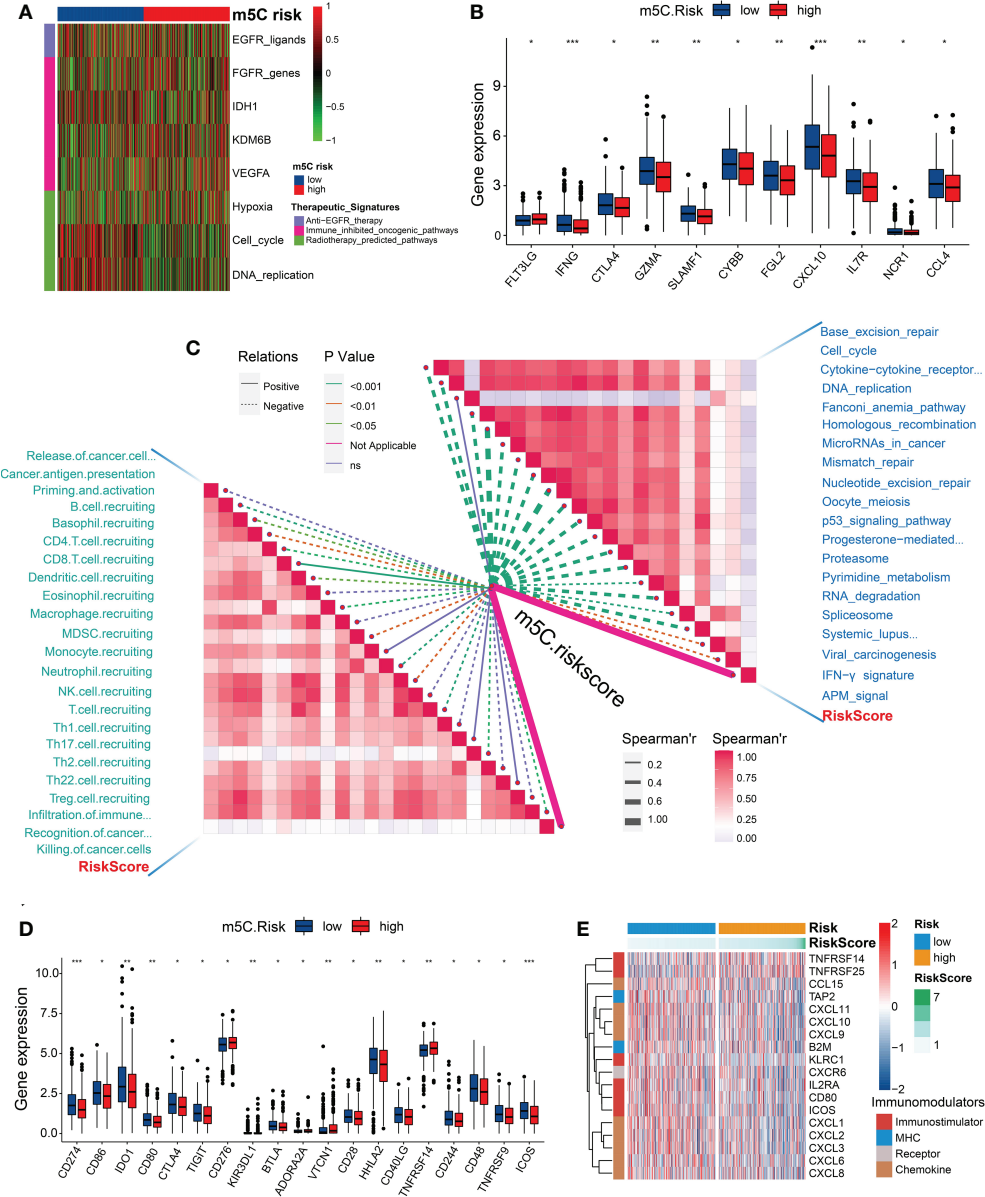
Correlation between the m5C risk score and immune phenotypes. **(A)** Heatmap showing the associations between the m5C score and the enrichment scores of several therapeutic signatures. **(B)** Differences in the expression levels of the immune effector genes between the two m5C score groups. **(C)** Spearman’s correlation analysis of the m5C score with the activities of cancer immunity cycles (left) and immune-related pathways analyzed by the ssGSEA (right). **(D)** Differences in the expression levels of immune checkpoint genes between the two m5C score groups. **(E)** Heatmap showing the significant differential expression of immunomodulators between the two risk groups. *P < 0.05, **P < 0.01, ***P < 0.001.

We investigated the existence of immune heterogeneity in different m5C risk groups. The correlation analysis between the m5C score and enrichment scores of the therapeutic signatures demonstrated that the CRC patients with low m5C score may have benefited from radiotherapy (**Figure 6A**). Common immune effector genes, including *IFNG*, *CTLA4*, *GZMA*, *SLAMF1*, *CYBB*, *FGL2*, *CXCL10*, *IL7R*, *NCR1*, and *CCL4*, were all highly expressed in the low-risk subgroup (**Figure 6B**), and the TIICs, such as activated CD8 and CD4 T cells, effector memory CD8 T cells, type 2 T helper (Th) cells, eosinophils, γδT cells, and neutrophils, were significantly enriched in the low-risk subgroup (**Supplementary Figure 5**). Additionally, the m5C score was negatively associated with the activities of many critical anticancer immunity cycles, including CD8 T cell recruitment; B-cell recruitment; cancer antigen presentation, priming and activation; myeloid-derived suppressor cell recruitment; neutrophil recruitment; natural killer cell recruitment; and Th1 cell recruitment (**Figure 6C**; **Supplementary Table 14**).

As expected, the m5C score was negatively correlated with ICB response-related pathways, including base-excision repair, antigen-processing machine signaling, the interferon-γ signature, spliceosome, RNA degradation, proteasome, and the p53 signaling pathway (**Figure 6C**). However, several common immune checkpoint genes, such as *CD274 (PD-L1)*, *CD86*, *CD80*, *CTLA4*, *IDO1*, *TIGIT*, *KIR3DL1*, *BTLA*, *CD28*, *HHLA2*, *CD40LG*, *CD244*, *CD48*, *TNFRSF9*, and *ICOS* (**Figure 6D**), were highly expressed in the low-risk subgroup. Furthermore, the genes associated with immunomodulation, including *CCL15*, *TAP2*, *CXCL11*, *CXCL10*, *CXCL9*, *B2M*, *KLRC1*, *CXCR6*, *IL2RA*, *CD80*, *ICOS*, *CXCL1*, *CXCL2*, *CXCL3*, *CXCL6*, and *CXCL8*, were significantly upregulated in the low-risk subgroup (**Figure 6E**; **Supplementary Table 15**).

**Figure 7:**
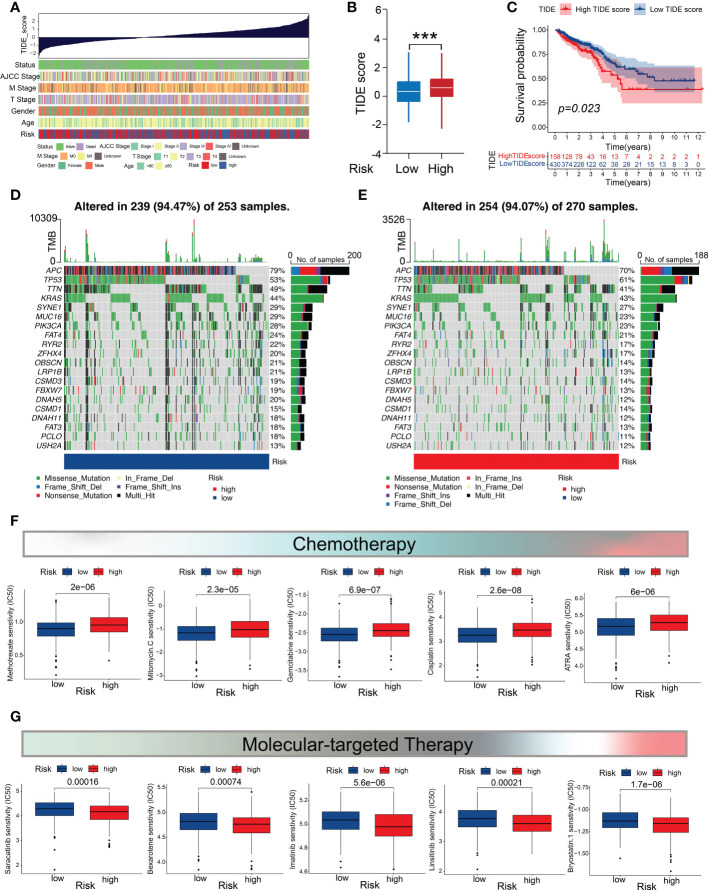
Mutation profiles and drug-susceptibility analysis. **(A)** Correlations between the TIDE scores and clinicopathological features (survival status, TNM stage, gender, age, and m5C score subtypes). **(B)** Comparison of the TIDE score between the two m5C score subgroups. **(C)** Survival analysis of the CRC patients in the high- and low-TIDE-score groups. **(D, E)** Waterfall chart depicting the somatic mutation landscapes in the low- and high-m5C-score groups. **(F)** Relationship between the m5C score and chemotherapeutic sensitivity. **(G)** Association between the m5C score and targeted treatment sensitivity. ***P < 0.001.

A higher TIDE prediction score has been confirmed to be associated with tumor escape from immune surveillance and worsened ICB response (35). In the present study, we found that patients with CRC in the low-risk subgroup exhibited a lower TIDE score than those in the high-risk subgroup (**Figures 7A, B**; **Supplementary Table 16**), whereas the patients with a high TIDE score had significantly worse prognoses than those with a low TIDE score (**Figure 7C**). These findings indicate that the low m5C score is associated with an inflammatory phenotype.

### Mutation profiles and drug-susceptibility analysis

Genomic mutations are considered to be the driving force of tumor malignancy. Therefore, we investigated and visualized the distribution of 20 somatic mutations between the two risk groups. The most frequently-mutated genes in the CRC population were *APC* and *TP53* (**Figures 7D, E**). Notably, the mutation frequencies of the most genes (19/20) were higher in the low-m5C-score subgroup (**Figures 7D, E**). We then investigated whether the m5C score could provide accurate guidance for precision treatments by assessing the differences in anticancer drug sensitivity between the low- and high-risk subgroups for identifying potential CRC individualized therapy modalities. The IC_50_ values demonstrated that the CRC patients with low m5C score exhibited higher level of sensitivity to common chemotherapeutic drugs, such as methotrexate, mitomycin C, gemcitabine, cisplatin, camptothecin, and all-trans retinoic acid (**Figure 7F**), whereas those with high m5C score showed higher level of sensitivity to several targeted drugs, including saracatinib, bexarotene, bryostatin 1, imatinib, and linsitinib (**Figure 7G**). These results demonstrate that the m5C score may contribute to identifying effective antitumor agents and precision medicine therapies for CRC treatment.

### Construction and performance validation of the m5C score-based nomogram

**Figure 8:**
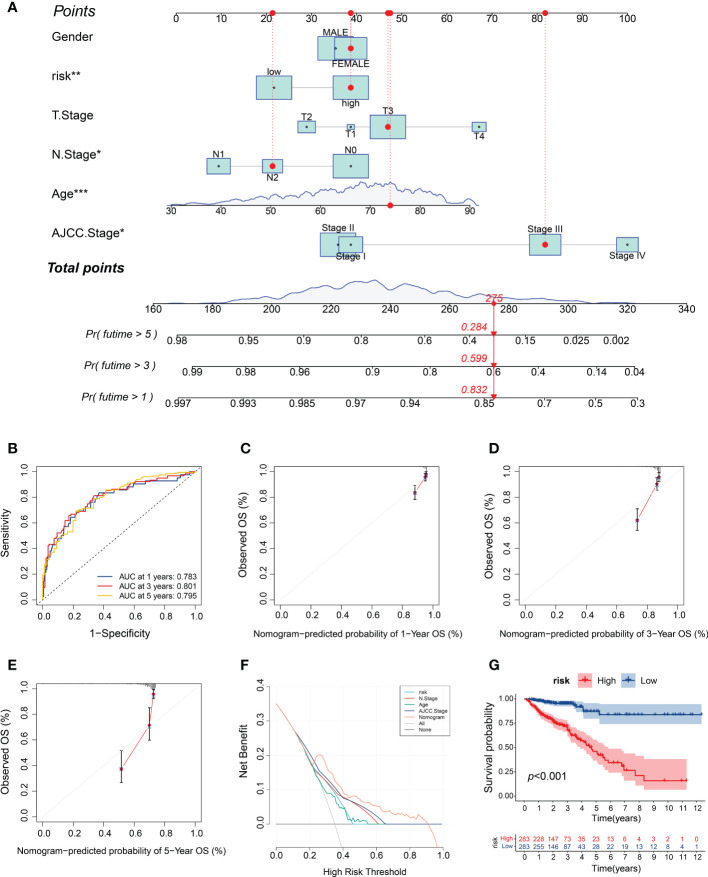
Construction and validation of the m5C score-based nomogram. **(A)** Development of the nomogram for predicting the 1-, 3‐ or 5‐year OS of CRC patients. **(B)** ROC curves for the nomogram for predicting the 1-, 3-, and 5-year OS. **(C–E)** Calibration plots of the nomogram for predicting the 1-, 3- and 5-year OS. **(F)** DCA for the nomogram assessing clinical utility. **(G)** Kaplan–Meier survival curves on the basis of the m5C score calculated using the nomogram. *P < 0.05, **P < 0.01, ***P < 0.001.

To provide clinicians with a quantitative method for predicting CRC prognoses, we constructed a nomogram that integrates the risk model and clinical variables, including gender, age, depth of tumor invasion (T stage), lymph node metastasis (N stage), and AJCC stage (**Figure 8A**). We subsequently validated the predictive accuracy of the nomogram by measuring the AUC and performing calibration. The ROC analysis revealed AUC values of 0.783, 0.801, and 0.795 for the prediction of the 1-, 3-, and 5-year overall survival (OS), respectively (**Figure 8B**), thus demonstrating the predictive ability of the nomogram. The calibration plot for the nomogram predicting 1-, 3-, and 5-year OS demonstrated good performance relative to that of an ideal model using TCGA sets (**Figures 8C–E**). Moreover, the DCA graphically demonstrated that the nomogram exhibited greater clinical usefulness and net benefit than the other models, indicating the powerful predictive ability for clinical application (**Figure 8F**). Based on the nomogram, we stratified the patients with CRC into high- or low-risk subgroups based on the median risk score. The survival analysis confirmed that the patients in the high-risk subgroup showed worse prognoses than those in the low-risk subgroup (**Figure 8G**). These results suggest that the m5C score-based nomogram represents a more accurate and reliable predictive model than conventional staging systems.

### External validation of the m5C score using GEO CRC and independent cohorts

**Figure 9:**
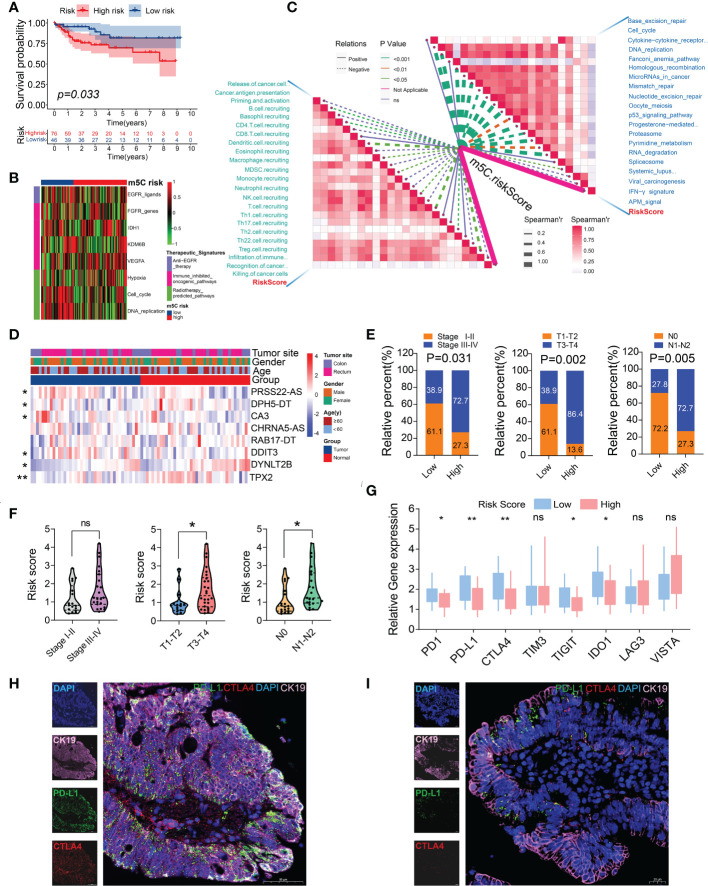
External validation of the m5C score using a GEO CRC dataset (GSE38832) and an independent CRC cohort. **(A)** Kaplan–Meier survival curve for patients with low and high m5C scores. **(B)** Heatmap of the associations between the m5C score and the enrichment scores of several therapeutic signatures in the GSE38832 dataset. **(C)** Spearman’s correlation analysis of the m5C score with activities of cancer immunity cycles (left) and immune-related pathways using the GSE38832 dataset. **(D)** Heatmap of the eight m5C-related risk gene profiles in 40 pairs of CRC tumor tissues and adjacent normal tissues. **(E)** Histogram showing the ratio of the AJCC, T, and N stages between the low- and high-risk groups. **(F)** Relationships between the m5C risk scores and clinicopathological characteristics of 40 CRC patients in our cohort. **(G)** The relative mRNA expression levels of several immune checkpoint genes were examined using RT-qPCR. **(H, I)** PD-L1 and CTLA4 expression was detected using immunofluorescence between the CRC patient samples in the low- (left) and high- (right) m5C-score groups. *P< 0.05, **P< 0.01, ns, no significant.

We verified the reliability of the m5C-based risk model using a GEO CRC cohort and 40 patients with CRC from our center. Consistent with the analysis results of the dataset from TCGA, the patients with CRC from the GSE38832 cohort and those in the high-m5C-score group showed worse prognoses than those in the low-m5C-score group (**Figure 9A**). The correlation analysis of the therapeutic signatures indicated similar treatment prediction results in the TCGA-CRC cohort (**Figure 9B**). The m5C score, which was also consistent with the aforementioned results, was negatively associated with most of the ICB response-related pathways and activities of many anticancer immunity cycles (**Figure 9C**). Furthermore, TIICs, including activated CD8 and CD4 T cells, effector memory CD8 T cells, and type 17 Th cells, were enriched in the low-m5C-score subgroup, whereas the infiltrating levels of pro-tumor immune cells (plasmacytoid dendritic cells) were significantly higher in the high-m5C-score group (**Supplementary Figure 6A**). The patients with CRC at AJCC stages III–IV showed significantly higher levels of enrichment in the high-risk subgroup than in the low-risk subgroup (**Supplementary Figure 6B**).

To further confirm the clinical significance of the risk model, the expression levels of eight m5C-related risk genes were determined in 40 pairs of CRC tumor tissues and corresponding normal tissues. The RT-qPCR analysis demonstrated the expression profiles of these genes, which were visualized using a heatmap. Six m5C-related risk genes were differentially expressed between the cancer tissues and adjacent normal tissues (**Figure 9D**; **Supplementary Table 17**). The 40 patients with CRC were stratified into two subgroups according to the risk formula. The results indicated that the m5C risk score was significantly correlated with the AJCC stage, T stage, and N stage (**Figure 9E**). In addition, the patients in the T3-T4 or N1-N2 group showed higher risk scores than those in the T1-T2 or N0 group (**Figure 9F**). The patients in the low-risk group exhibited higher levels of immune checkpoint genes, including *PD-1*, *PD-L1*, *CTLA4*, *TIGIT*, and *IDO1* (**Figure 9G**). Consistent with the analysis results of the data from TCGA, the immunofluorescence results revealed that the patients in the low-risk group showed a higher percentage positivity and cell counts of PD-L1 and CTLA4 than those in the high-risk group (**Figures 9H, I**). The results of the external validation further confirmed the effectiveness of using the m5C score as an indicator of CRC prognoses and the relative immune response.

## Discussion

Cancer immunotherapy represents a newly-emerging and rapidly-growing field in precision medicine for clinical applications and research settings (36). Significant breakthroughs in cancer immunotherapy have allowed for a broader understanding of the influence of the tumors genetic landscapes on the treatment sensitivity of immunotherapy as a critical cornerstone for the implementation of individualized cancer therapies (37). Currently, several promising immunotherapies, such as those involving oncolytic viruses, immune checkpoint inhibitors (ICIs), chimeric antigen receptor T cells, and cancer vaccines, represent alternative strategies for treating various cancers (38). Compared with the standard treatments, immunotherapy utilizes and manipulates the immune system of the patient to attack malignant cells, thereby enabling innate and adaptive immune factors to identify cancer cells and potentially initiate tumor-specific immune responses (39–41). Cancer immunotherapy has achieved a remarkable level of efficacy, especially in treating solid organ tumors and hematological malignancies (42, 43). CRC, however, is complex and present a high degree of TME heterogeneity, which introduces major variability in immunotherapeutic efficacy. Thus, investigating TME heterogeneity may facilitate improved prognostic predictions and precise treatment modalities for CRC. In this study, we identified two distinct m5C modification patterns in CRC, each being associated with different biological functions, immunological properties, and prognoses. To the best of our knowledge, this study presents the most comprehensive analysis of m5C regulators. We further developed an m5C risk-score model to quantify patient m5C subtypes and independently validated this model using two CRC cohorts.

As the most predominant epigenetic modification, RNA methylation plays an indispensable biological role in malignant transformation and cancer progression. Accumulating evidences have confirmed the regulatory effects of m6A RNA modifications in the TME and innate immunity of CRC (44–46). Another well-studied RNA modification is m5C, which is a common epigenetic modification that is widely involved in cancer initiation and progression (19–24). SUMO-2/3-modified NSUN2 reportedly promotes the progression of gastric cancer by regulating m5C mRNA methylation (23). Chen et al. developed a single-nucleotide resolution map of m5C modifications in human urothelial carcinoma of the bladder and identified high m5C methylation levels in oncogenes (19). Mechanistically, the m5C methyltransferase NSUN2 and the m5C reader YBX1 drive cancer progression by targeting the m5C methylation site of the 3′ untranslated region of HDGF (19). In esophageal squamous cell carcinoma, NSUN2 and LIN28B enhance the stability of GRB2 mRNA in an m5C-dependent manner, thereby facilitating cancer emergence and progression (24). However, comprehensive analyses of m5C RNA modifications and the TME in CRC has not yet been reported.

In this study, high-throughput sequencing and the HPA revealed imbalances in the expression levels of m5C writers, readers, and erasers. Theoretically, these imbalances may lead to aberrant m5C modification patterns, ultimately resulting in CRC tumorigenesis and progression. Moreover, we found that these m5C genes were highly interconnected and formed a tight network of molecular interactions. The cluster analysis identified two independent m5C modification patterns based on 18 m5C regulators. The survival analysis revealed significantly worse prognoses for the patients with CRC in m5C cluster B compared to those in m5C cluster A. Additionally, we observed significantly-different TME features between the two clusters, with cluster-specific DEGs also being associated with immune-related biological functions and cancer-related pathways.

Conventional radiotherapy and chemotherapy have yielded limited therapeutic efficacy in patients with advanced CRC. To date, three ICIs have been approved by the US Food and Drug Administration for the treatment of CRC, including the monoclonal antibodies pembrolizumab and nivolumab, which target PD-1, and ipilimumab, which targets CTLA-4 (47). Regardless of the significant advances in immunotherapy for cancer, substantial prognostic heterogeneity remains prevalent in CRC, which highlights the crucial contribution of the TME to CRC-targeted immunotherapy. We, therefore, established a robust risk-score model on the basis of the m5C-related genes. The results indicated that the m5C score not only accurately predicted the prognoses of the patients with CRC but also served as an efficient predictor of their immunotherapeutic response. Importantly, the patients in the high- and low-m5C-score groups presented distinct clinicopathological features, mutation patterns, immune checkpoint characteristics, immune cell infiltration, and drug sensitivity. Clinical trials of several ICIs that target PD-L1, CTLA-4, TIGIT, IDO1, TIM-3, LAG-3, and VISTA are currently underway for advanced solid cancers, including CRC (48).

In the high-m5C-score group, the expression levels of common immune checkpoint genes, including *PD-L1*, *IDO1*, *CTLA-4*, and *TIGIT*, were significantly lower than those in the low-m5C-score group. Moreover, the m5C score was negatively associated with the activities of many anticancer immunity cycles and immunomodulators, such as CXCR6, CXCL9, CXCL10, and CXCL11, which are of crucial importance for the infiltration of anticancer TIICs. Increasing studies have assessed the contribution of cytotoxic cells, especially CD8 T cells (49); however, recent studies have revealed that CD4 T cells exert their antitumor effects by directly inhibiting the tumor cell cycle (50–52). Indeed, γδT cells are able to recognize and kill CRC cells in a major histocompatibility complex-unrestricted manner (53), thereby inhibiting tumor progression. In the present study, we found that infiltrating levels of CD4 T cells, CD8 T cells, and γδT cells were significantly higher in the low-m5C -score subgroup, indicating their positive functions in CRC. The results further demonstrated that the high m5C score reflected a non-inflammatory phenotype, whereas the low m5C score reflected an inflammatory phenotype.

Targeted and ICI therapies are recommended as the mainstream treatment options for advanced mCRC. Despite the progress in the selection of molecular- targeted drugs based on specific gene loci for the individualized treatment of CRC, immunotherapeutic benefits have only been observed in a small percentage of patients with hypermutated MSI-high/deficient mismatch repair (MSI-H/dMMR) CRC (~15% of patients with localized CRC and 4% of patients with mCRC) (54). Despite there being strong evidence that ICI therapies have yielded potent and persistent effects in patients with MSI-H/dMMR CRC, some patients still fail to respond to immunotherapy or respond only partially (55). The optimal treatment regimen for specific patients remains difficult to determine; therefore, identifying predictive biomarkers with a higher degree of accuracy is necessary. We found that the m5C score may represent a biomarker that is capable of guiding clinical decision-making and facilitating personalized precision treatments for patients with CRC.

Our results further indicated that the CRC patients with high m5C risk score were sensitive to molecular-targeted drugs, with the findings suggesting that the m5C score may be useful in guiding the personalization of treatments for patients. Additionally, we established a nomogram model by incorporating clinical risk factors and the m5C score, which further improved prognostic performance. These results strongly indicate that the application of the m5C risk score for the prognostic stratification of CRC has great potential and could lead to better understanding of the molecular mechanisms underlying CRC, which would further contribute to developing improved therapeutic strategies.

Despite its promising findings, this study, nonetheless, had several limitations. All of the transcriptomic and expression data of the patients with CRC were extracted and analyzed from public databases (TCGA and GEO). Furthermore, this study utilized a retrospective analysis, which can introduce inherent selection bias. To address this limitation in future studies, we will cooperate with the Hubei Provincial Human Genetic Resources Collection Center and Hubei Key Laboratory of Intestinal and Colorectal Diseases to establish our own large-sample dataset for further evaluation and validation of our proposed model. Additionally, although we highlighted the use of m5C modification patterns for predicting CRC TME statuses and prognoses, we did not identify the associated molecular mechanisms. In the future, we will evaluate the biological functions associated with m5C modifications and those related genes to the regulation of the immune microenvironment, as well as the precise mechanisms underlying the m5C regulators in CRC based on the results of this study.

## Conclusions

In summary, these findings reveal the crucial role of m5C modification patterns in the regulation of the TME in CRC. The comprehensive analysis indicated that the novel m5C risk scores reflect the distinct prognostic signatures, clinicopathological characteristics, immunological phenotypes, and therapeutic opportunities that may promote the applications of precision medicine for patients with CRC.

## Data availability statement

The original contributions presented in the study are included in the article/**Supplementary Material**. Further inquiries can be directed to the corresponding authors.

## Ethics statement

The studies involving human participants were reviewed and approved by Ethical Committee of Zhongnan Hospital of Wuhan University. The patients/participants provided their written informed consent to participate in this study.

## Author contributions

CBX, XYQ, ZJH, and HYT designed the study; CBX, XYQ, ZJH, and HYT carried out the most experiments. TSH and ZX contributed to patient samples and clinical information. CBX, XYQ, ZJH, and HYT performed bioinformatic analysis. ZX and TSH conducted the statistical analysis. CBX and HYT wrote the manuscript. JCQ, RXH, FLF, XXY, and CQJ revised the paper. All authors read and approved the final manuscript.

## Funding

This work was supported by Enginerring construction project of improving diagnosis and treatment ability of difficult diseases (oncology) (Grant No. ZLYNXM202012), Joint Foundation of Health Commission of Hubei Province (Grant No. znpy2019086), and Wu Jieping Medical Research Foundation (No.320.6750.2021-11-8).

## Acknowledgments

The authors would like to thank Editage (http://www.editage.co.kr) for the professional language editing service.

## Conflict of interest

The authors declare that the research was conducted in the absence of any commercial or financial relationships that could be construed as a potential conflict of interest.

## Publisher’s note

All claims expressed in this article are solely those of the authors and do not necessarily represent those of their affiliated organizations, or those of the publisher, the editors and the reviewers. Any product that may be evaluated in this article, or claim that may be made by its manufacturer, is not guaranteed or endorsed by the publisher.
