# Supplementary figures and images for: m5C regulator-mediated modification patterns and tumor microenvironment infiltration characterization in colorectal cancer: One step closer to precision medicine
Source: Front Immunol. 2022 Dec 1;13:1049435. doi: 10.3389/fimmu.2022.1049435 (PMC9751490; doi:10.3389/fimmu.2022.1049435)

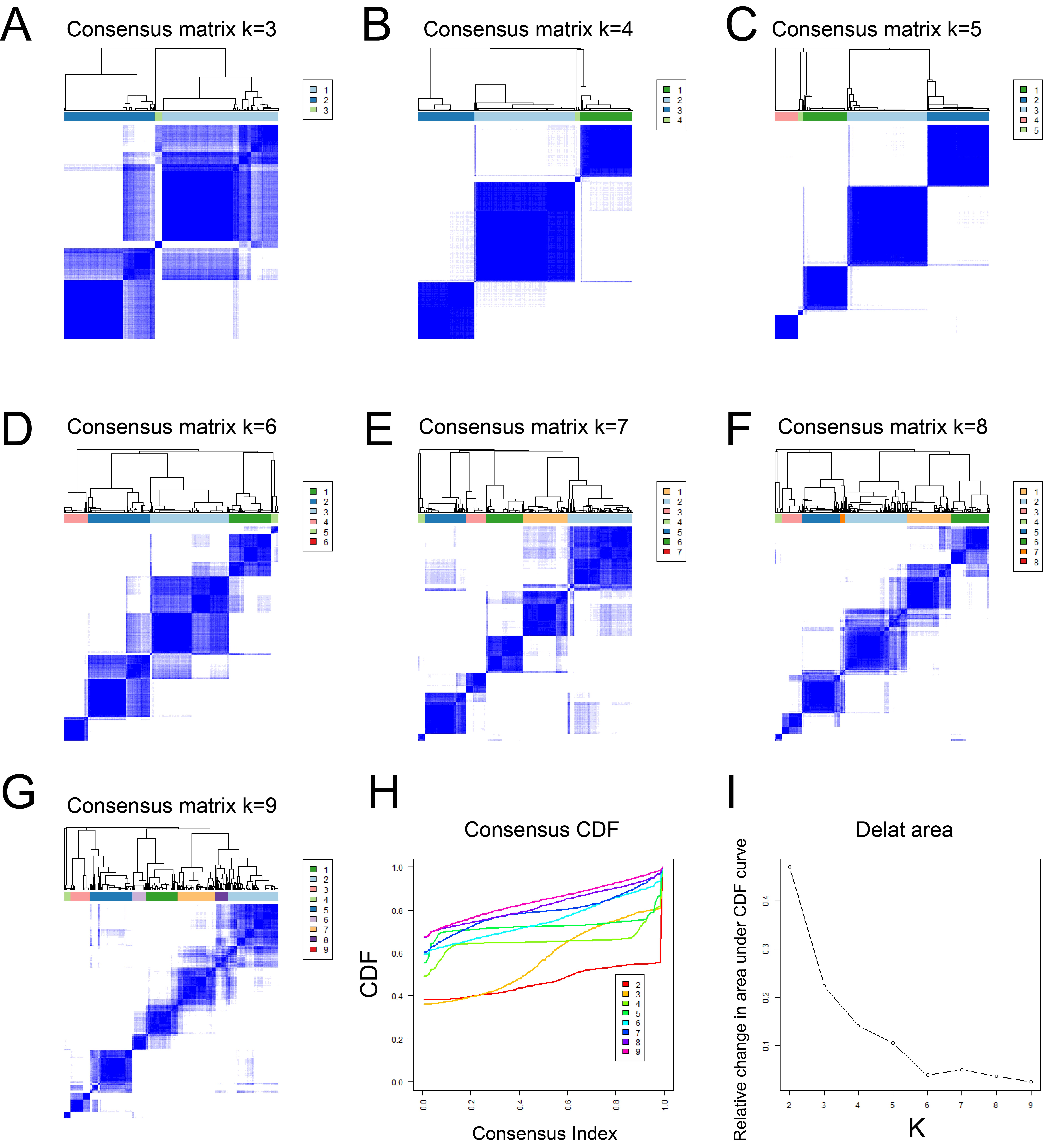

Supplement: Supplementary file 3 [file Image_3.jpeg]

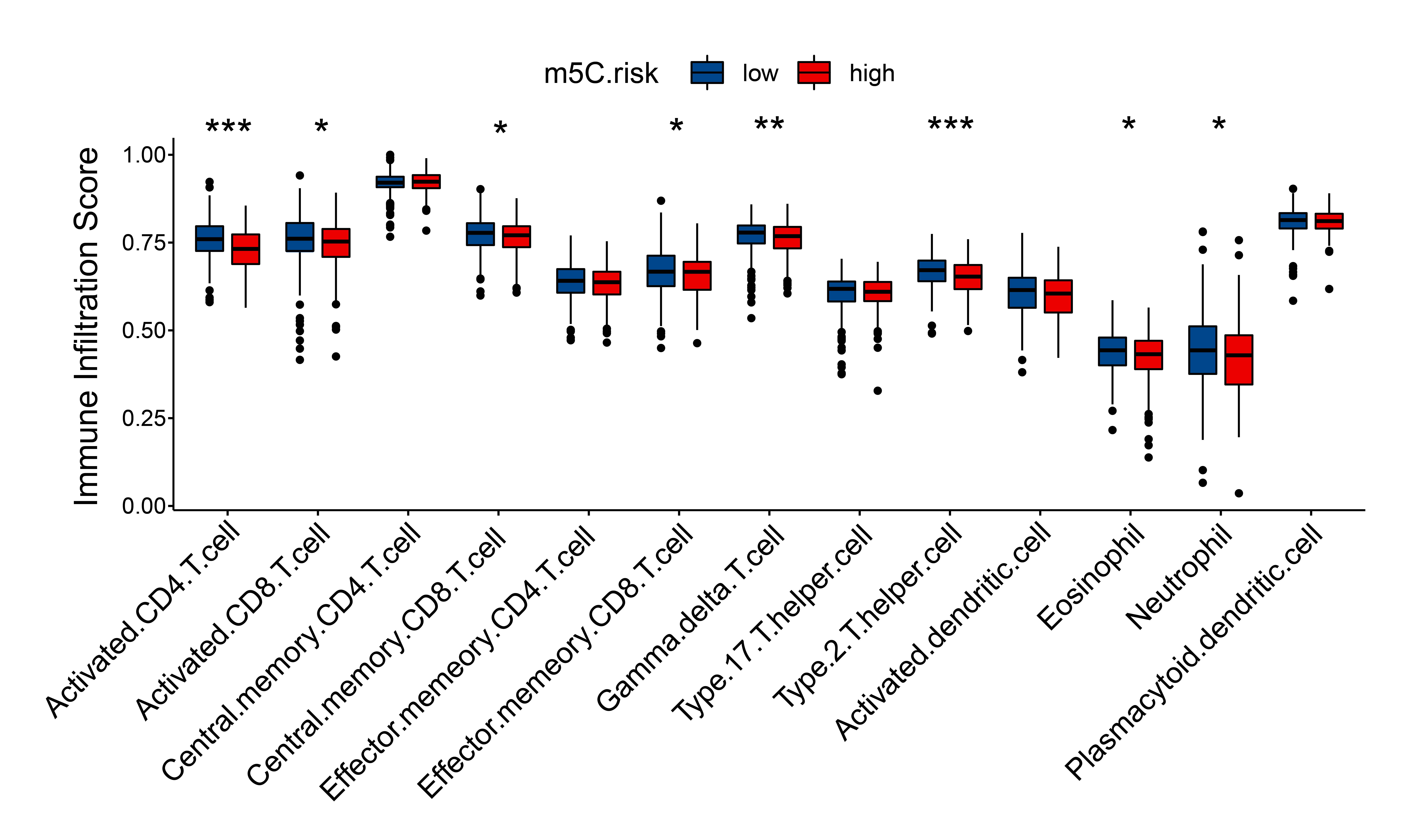

Supplement: Supplementary file 5 [file Image_5.jpeg]

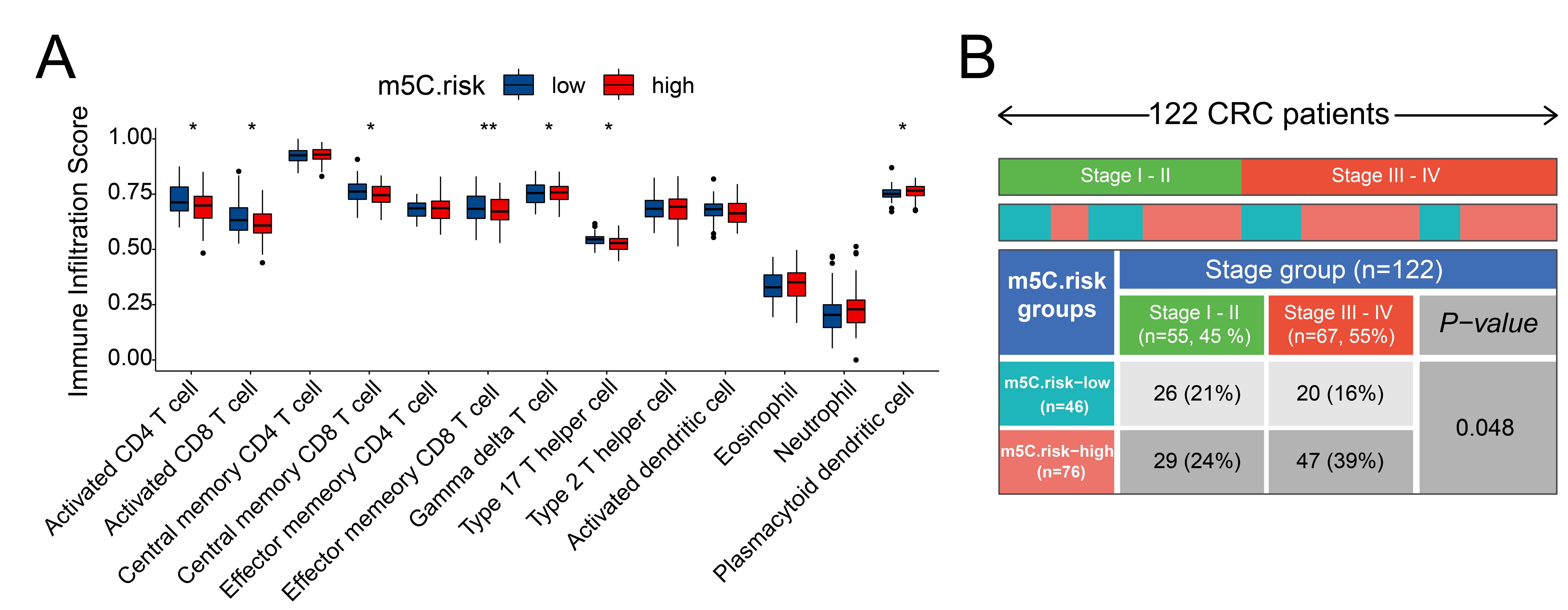

Supplement: Supplementary file 6 [file Image_6.jpeg]
